# Ipsilateral Zoster Ophthalmicus Post COVID-19 Vaccine in Healthy Young Adults

**DOI:** 10.7759/cureus.16725

**Published:** 2021-07-29

**Authors:** Kundana Thimmanagari, Sindhusha Veeraballi, Dawn Roach, Bader Al Omour, Jihad Slim

**Affiliations:** 1 Internal Medicine, Saint Michael's Medical Center, Newark, USA; 2 Medical Education, Saint Michael's Medical Center, Newark, USA; 3 Infectious Diseases, Saint Michael's Medical Center, Newark, USA

**Keywords:** herpes zoster, zoster ophthalmicus, covid-19 vaccine, hhv3, herpes zoster reactivation

## Abstract

Herpes zoster ophthalmicus (HZO), which is an ophthalmological emergency, accounts for 10%-20% of all Herpes zoster (HZ) cases. HZ infection in COVID-19 vaccinated individuals who are immunocompetent can be attributed to vaccine-induced immunomodulation allowing the varicella-zoster virus (VZV) to escape from the dorsal root ganglia. Another theory is similar to immune reconstitution syndrome (IRS). HZ infection in a young immunocompetent individual is a rare entity. As per our literature review, only four cases have been reported thus far. We are reporting two cases of two young individuals with no known risk factors who developed ipsilateral HZO after receiving the COVID-19 vaccination. The increasing incidence of HZ cases post COVID-19 vaccine indicates that this is not a mere coincidence. Awareness must be created among physicians, as well as the general population, for early recognition and early antiviral usage, which can halt the progression of the disease and thus prevent debilitating complications.

## Introduction

Reactivation of varicella-zoster virus (VZV) in the ophthalmic division of trigeminal nerve manifests as an ophthalmologic emergency termed herpes zoster ophthalmicus (HZO). HZO accounts for 10%-20% of all herpes zoster (HZ) cases. Reactivation is attributed to a decline in T-cell immunological response due to a variety of reasons such as advanced age, immunosuppressive medication, malignancy, chemotherapy, and HIV/AIDS [1]. We report two cases of HZO in healthy, middle-aged men without any physical or emotional stressors approximately one week after receiving a COVID-19 vaccine. Interestingly, in both cases, the pain and eruption of lesions occurred ipsilateral to the arm they received their COVID-19 vaccines in.

## Case presentation

Case 1

A 42-year-old male with a past medical history of asthma presented to the emergency room with complaints of worsening painful lesions on the left side of his forehead and blurred vision. He stated that he received Johnson and Johnson COVID-19 vaccine on his left arm recently, after which he experienced generalized weakness for two days. One week after receiving the vaccine, he noticed skin lesions that started as small pimples on the left side of his forehead that were associated with burning pain and itching. The lesions rapidly progressed, involving the left side of the scalp, the tip of the nose, and the left upper eyelid. Associated symptoms were watery discharge from the eyes and blurred vision. He denied having any other complaints. On further questioning, he admitted to a history of chickenpox at the age of 10. He denied any sick contacts, recent emotional or physical stress, recent travel, or steroid usage in the past one year.

**Figure 1:**
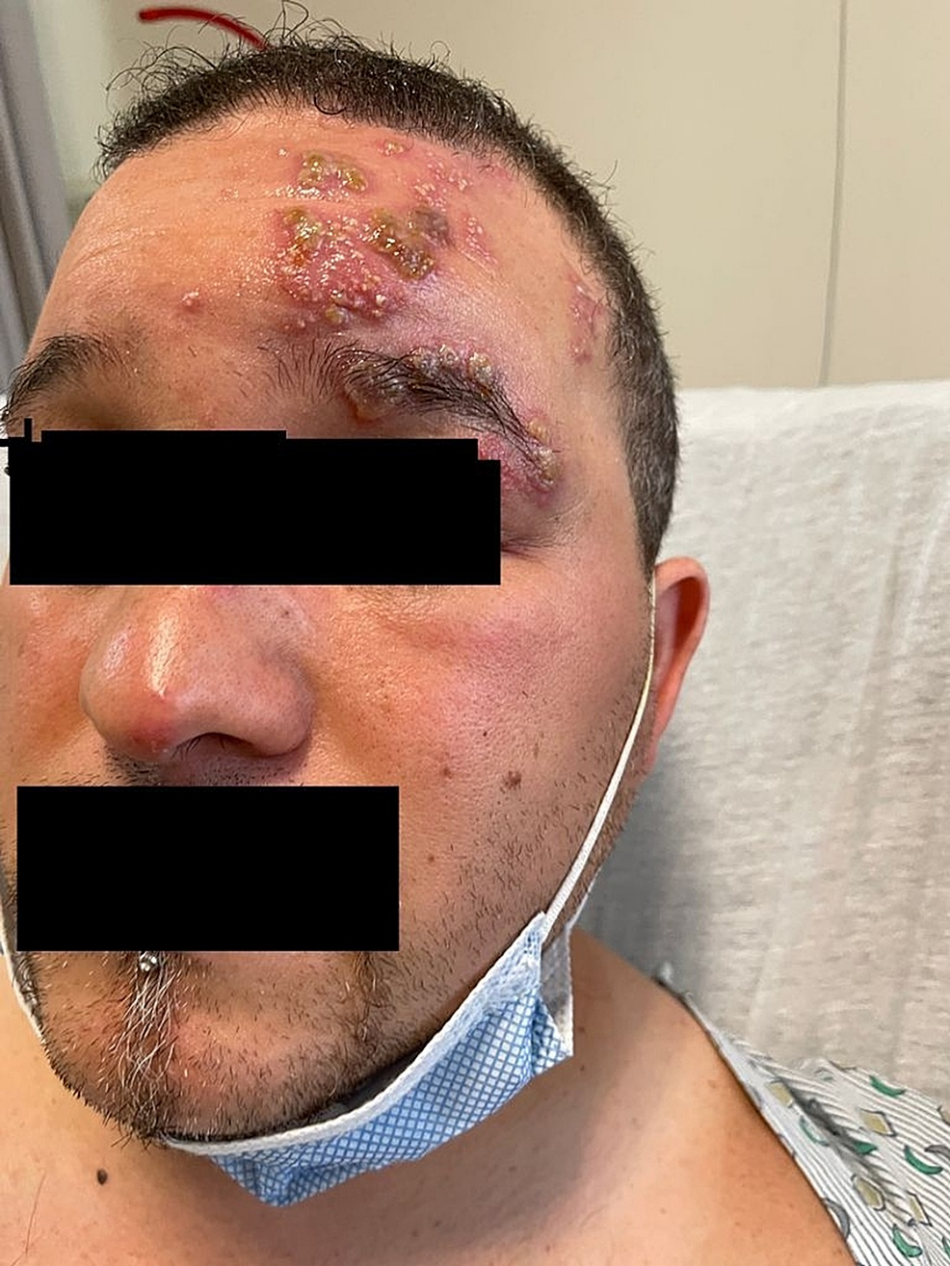
Erythematous grouped vesicles on the left side of the face

Vitals: BP 151/89, RR 18, HR 93, afebrile. On physical examination, grouped vesicles with the erythematous base were observed on the left side of his forehead, left upper eyelid, left side of the scalp, and the tip of his nose (Figure 1). The left eyelid looked edematous with conjunctival injection and watery discharge, with no visible corneal endothelial decompensation. The restricted ocular movement was noticed due to edema of his upper eyelid. Visual acuity was also limited. Labs: White count 5.50 cells/microliter (normal: 4,500-11,000), hemoglobin 15 g/dL (normal: 13.5-17.5), and platelets 274K cells/microliter (normal: 150k-450k). Based on the clinical examination findings, the diagnosis of HZO was made. He was started on systemic antiviral and antiviral eye drops, which halted the progression of vesicles and improved his vision.

Consent from a patient was taken to use his image for the journal publication.

Case 2

A 49-year-old male with a past medical history of bipolar disorder, schizophrenia, and hyperlipidemia presented to the emergency room with worsening burning pain on the right side of the forehead and blurred vision. He has no history of shingles in the past. On further questioning, he stated that he received the first dose of the Moderna vaccine one week ago in his right arm. He denied having any other complaints. He works as a caretaker and lives with his parents. Furthermore, he denied having any stressors at work or home.

Vitals: BP 146/84, RR 20, HR 88, Temp 100F. Labs: White count 6.70 cells/microliter (normal: 4,500-11,000), hemoglobin 16.9 g/dL (normal: 13.5-17.5), and platelets 198K cells/microliter (normal: 150k-450k). Clinical examination revealed grouped erythematous papules and vesicles on the right side of the forehead (i.e., V1 dermatome) and edema of the right upper eyelid. Based on the clinical examination, the diagnosis of HZO was made. He was started on systemic antiviral therapy with an improvement of his symptoms.

## Discussion

VZV is a DNA virus from the family herpesvirus, also known as HHV3. It causes two clinical conditions. The primary infection causes chickenpox. The secondary infection causes varicella zoster, which is due to the reactivation of latent VZV. VZV reactivation occurs when there is downregulation of major histocompatibility complex class 1 resulting in inhibition of interferon response which acts as an antiviral, thus triggering the viral replication [1]. Examples of risk factors that are associated with this downregulation of immune response are stress, trauma, advanced age, HIV, malignancy, chronic kidney disease, and liver disease. Typically, VZV infection presents with a rash and acute neuropathic pain [2]. The rash is usually confined to the ipsilateral dermatome. However, a disseminated presentation involving more than one dermatome has been seen, especially in severely immunosuppressed patients. The characteristic lesions are grouped vesicles or bullae, which become pustules in several days. The most common dermatomes involved are thoracic and lumbar. Life-threatening involvement of the ophthalmic branch of the trigeminal nerve has also been identified, causing HZO. The incidence rate of HZO is approximately 10%-20% of HZ infections [2]. Common presenting symptoms of HZO are headache, rash, conjunctivitis, scleritis, keratitis, retinal necrosis, and meningoencephalitis [2]. Early recognition and treatment with an antiviral is the mainstay of management and can prevent vision loss and life-threatening complications. It is recommended individuals greater than 50 years of age who are immunocompetent be vaccinated against HZ.

A variety of dermatological reactions have been reported post COVID-19 vaccination [3]. Individuals infected with SARS-COV-2 have lymphopenia, which causes recurrence of HZ infection [4,5]. HZ infection in vaccinated individuals who are immunocompetent can be attributed to vaccine-induced immunomodulation allowing VZV to escape from dorsal-root ganglia. Another theory is similar to immune reconstitution syndrome (IRS), which is worsening of pre-existing infection seen in immunocompromised HIV patients. In IRS T-cells have been completely suppressed and once Antiretroviral therapy (ART) is initiated a robust increase in T-cells causing an acute inflammatory phase reaction begins [6]. HZ infection in a young immunocompetent individual is a rare entity, and thus far only four cases have been reported [7]. We are reporting two middle-aged individuals with no known risk factors who developed HZ post COVID-19 vaccination.

The website to report any adverse events is https://vaers.hhs.gov.

## Conclusions

Our case report emphasizes that HZ post COVID-19 vaccine is beyond coincidence. Awareness must be created among physicians, as well as the general public by reporting any associations with the COVID-19 vaccine and HZ to CDC. Early recognition and antiviral usage can halt the progression of the disease and thus prevent debilitating complications. The fear of HZ should not inhibit individuals from receiving the COVID-19 vaccine. Further studies must be conducted to completely understand the pathophysiology and construct preventive measures.
